# Accumulation of blood chromium and cobalt in the participants with metal objects: findings from the 2015 to 2018 National Health and Nutrition Examination Survey (NHANES)

**DOI:** 10.1186/s12877-022-03710-3

**Published:** 2023-02-03

**Authors:** Jinshen He, Jinfei Li, Song Wu, Jiaoju Wang, Qi Tang

**Affiliations:** 1 Department of Orthopaedic Surgery, the Third Xiangya Hospital of Central South University, Changsha, 410013 Hunan China; 2 Mathematics and Statistics School, Central South University, Changsha, 410000 Hunan China; 3 Department of Rheumatology and Immunology, the Second Xiangya Hospital of Central South University, Changsha, 410011 Hunan China

**Keywords:** Metal object, Cobalt, Chromium, NHANES

## Abstract

**Background:**

Chromium (Cr) and cobalt (Co) are the essential elements for producing metal implants, but might have potential health issues. The research on the correlation between metal implants and blood Cr and Co on a large population is still limited.

**Methods:**

National Health and Nutrition Examination Survey (NHANES) is a program of studies designed to assess the health status of Americans began in the early 1960s. The study was based on the NHANES database from two data collection years (2015–2016 and 2017–2018). The exposure variable of this study was whether the participants had metal objects in the body or not. The outcome variables were blood concentrations of Cr and Co. Age, body mass index, sex, race/ethnicity, income to poverty ratio, tap water behavior, shellfish/fish/tuna/salmon eating habits, level of education, smoking behavior, marital status, blood hemoglobin, and data collection years were included as confounding variables.

**Results:**

A total of 4412 participants, aged 40 years or older, were included in this analysis, consisting of the without metal objects group (*n* = 3150) and the metal objects group (*n* = 1262). Metal objects was positively correlated to the accumulation of blood Cr (β = 0.072, 95% CI: 0.043–0.102, *p* < 0.001) and blood Co (β = 0.079, 95% CI: 0.049–0.109, *p* < 0.001). However, the positive correlation of metal objects with blood Cr was only presented in women (β = 0.112, 95% CI: 0.074–0.151, *p* < 0.001), but not in men. Meanwhile, the positive relationship between metal objects and blood Cr/Co was not observed in the Asian subgroup.

**Conclusions:**

Blood Cr and Co concentrations were statistically higher in people with metal objects, but with race and sex differences.

**Level of Evidence:**

Level IV, cross-sectional study

**Supplementary Information:**

The online version contains supplementary material available at 10.1186/s12877-022-03710-3.

## Introduction

Metal objects such as artificial joints, pins, plates, or metal suture material are mainly considered treatment options for orthopedic diseases, including osteoarthritis [1, 2], fracture [3, 4], bone tumor [5], and others [6]. For orthopedics, as one of the ordinary metal objects, the artificial joint is to diminish pain, correct deformity, restore function, and improve patients’ quality of life. However, patients’ or their families’ concerns about the adverse effects of those implants have been raised for the possible accumulation of metal ions in the blood.

Humans can be exposed to metals in various ways. Drinking water and eating foods such as fish, smoking, geographical environment, and occupational exposures have been suspected as sources of heavy metals [7–9]. Chromium (Cr) and cobalt (Co) are the essential elements for producing metal implants, but might have potential health issues. One study based on 100 patients with metal implants has shown that serum Co ion level was elevated after metal-on-metal total hip arthroplasty [10]. A recent study on 51 patients also reported a relationship between increased serum Cr and Co levels and spinal implants [11]. The literature review based on 43 studies indicated blood concentration of Cr ranged between 0.5 and 2.5 μg/L, and Co ranged from 0.7 to 3.4 μg/L among those patients exposed to metal-on-metal implants [12–14] Meanwhile, percutaneous coronary intervention with Co-Cr coronary stents does not cause serum Cr and Co concentrations elevation based on 20 patients [6]. The controversial results might be due to the limitation of the sample size, so the research on the effect of metal implants on a large population is still needed.

Fortunately, the extensive data capabilities with thousands of participants in the United States National Health and Nutrition Examination Survey (NHANES) database resolve the data acquisition difficulty. Therefore, this study aimed to reveal the association between metal implants and the blood Co or Cr level (Co/Cr) in the population with different race/ethnicity and sex from NHANES. It was hypothesized that the blood Co/Cr in the participants with metal implants were significantly higher than those without metal implants.

## Materials and methods

### Study population

**Fig. 1:**
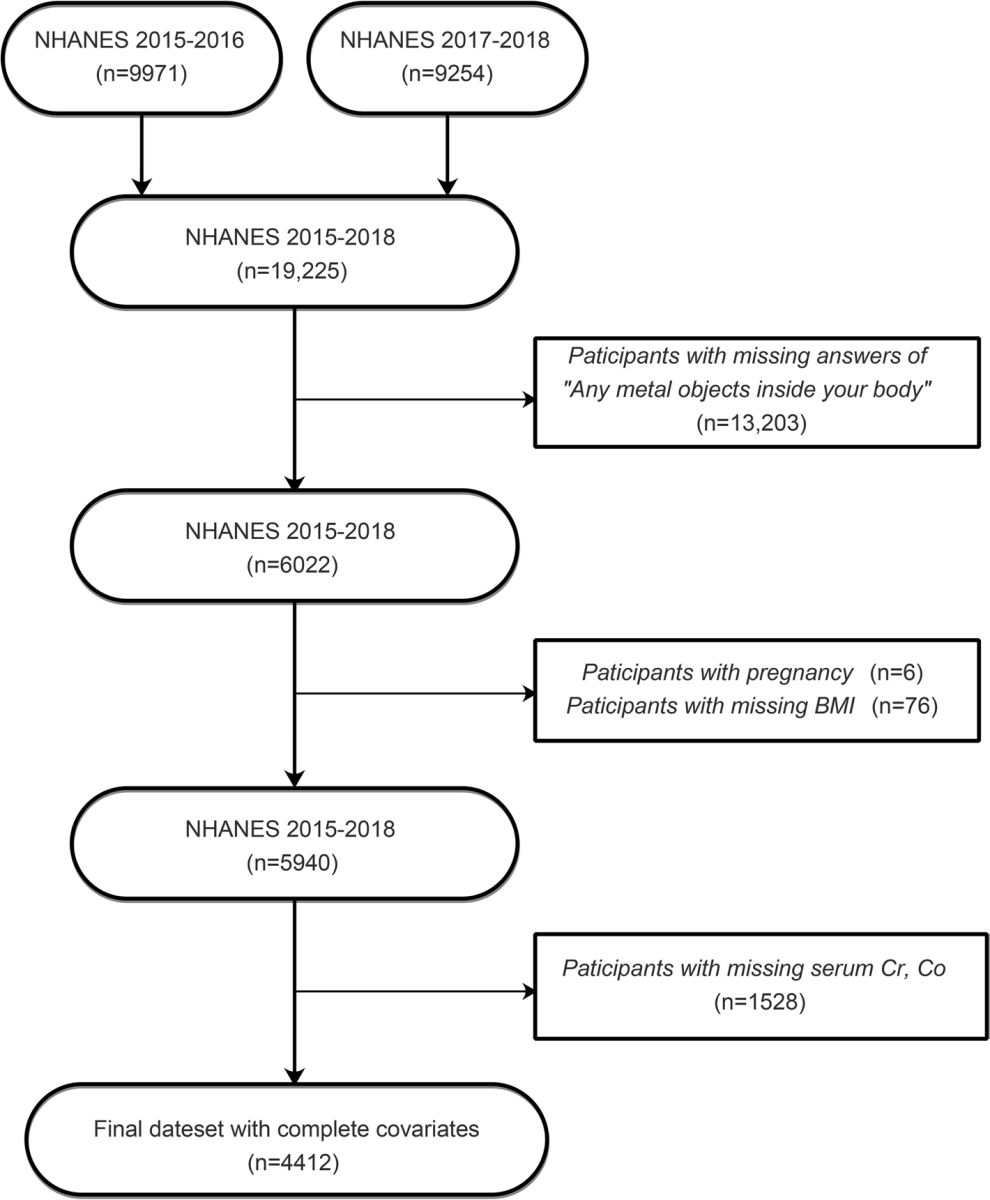
Flow chart of sample selection from the NHANES 2015–2018

The study population was based on the two data collection years of the NHANES database from 2015–2016 and 2017–2018 (Fig. 1). After exclusion of participants with missing metal objects information (*n* = 13,203), participants with pregnancy (*n* = 6), participants with missing body mass index (BMI) (*n* = 76), and participants with missing blood Cr/Co data (*n* = 1527), a total of 4412 participants were included in this analysis. Approval of this study was obtained from the ethics review board of the National Center for Health Statistics, and written consent was obtained from every participant.

### Variables

The exposure variable of this study was the participants with metal objects or not. Newly released in the data collection years of 2015–2016, the NHANES contained the question “Any metal objects inside your body?” in the Medical Conditions Data of the Questionnaire Data section (https://wwwn.cdc.gov/Nchs/Nhanes/2015-2016/MCQ_I.htm). The definition of metal objects in the question contained any artificial joints, pins, plates, metal suture material, or other types of metal objects in the body. The metal object should not be visible on the outside of the body or in the mouth. The interviewers aged 40 years and older were eligible to answer yes or no.

The outcome variable was the whole blood concentrations (ug/L) of Cr and Co, measured using inductively coupled plasma mass spectrometry [15]. The detailed description of the laboratory methods was noted in the Laboratory Method Files section (https://wwwn.cdc.gov/Nchs/Nhanes/2015-2016/CRCO_I.htm).

The following categorical variables were included in this analysis: sex (male or female), race/ethnicity, income to poverty ratio, tap water behavior, shellfish/fish/tuna/salmon eating habits, level of education, smoking behavior, marital status, and data collection years. In addition, the continuous covariates were also included: age, BMI, and blood hemoglobin. The detailed definitions of the covariates are available at https://wwwn.cdc.gov/nchs/nhanes/. Those confounders were evaluated using prior knowledge [16–19] and descriptive statistics from our cohort through the use of directed acyclic graphs (Supplement Fig. 1).

### Statistical analysis

The weighted χ2 test for categorical variables or linear regression model for continuous variables were applied to calculate the difference between the groups with or without metal objects. A multivariate linear regression model was applied to evaluate the association between metal objects and blood Cr/Co. The subgroup analysis was performed by stratified multivariate regression analysis. Furthermore, to detect trends, smooth curve fittings were used to address the relationship between age/BMI and blood Cr/Co in different groups. All analyses were performed with EmpowerStats software (version 3.0, X&Y Solutions, Boston, MA, USA) and the R Project for Statistical Computing (version 3.2.3, R Core Team), and *p* < 0.05 was deemed statistically significant. The R code of the modified ggplot2 package to draw smooth fitting curves was attached in the Supplement file 1.

## Results

A total of 4412 participants, aged 40 years or older, were included in this analysis, consisting of the without metal objects group (*n* = 3150) and with metal objects group (*n* = 1262), as shown in Table 1. There were statistically significant differences in baseline characteristics between the groups, except the BMI, income to poverty ratio, blood hemoglobin, data collection years, marital status (Table 1 and Supplement Table 1). Compared to the group without metal objects, participants were more likely to be older, Whites, smoke ≥ 100 cigarettes, drink tap water, and eat shellfish, fish, tuna, or salmon. 74.8% of participants with metal objects are non-Hispanic White, but the incidence is only 63.6% in the group with metal objects.

The results of the multivariate regression analyses were presented in Table 2. In the unadjusted model, metal objects were positively correlated to blood Cr accumulation (β = 0.074, 95% CI: 0.046–0.102, *p* < 0.001). After adjustment for confounders, this positive association was still present in minimally adjusted model (β = 0.076, 95% CI: 0.047–0.105, *p* < 0.001) and fully adjusted model (β = 0.072, 95% CI: 0.043–0.102, *p* < 0.001). Meanwhile, metal objects were positively correlated to the blood Co accumulation in all three models. Individuals with metal objects had a 0.079 ug/L greater blood Co than those without metal objects (Table 2).

On subgroup analyses, stratified by sex, reported in Table 3, the positive correlation of metal objects with blood Cr was only presented in women (β = 0.112, 95% CI: 0.074–0.151, *p* < 0.001), but not in men. The positive correlation of metal objects with blood Co remained in both men (β = 0.102, 95% CI: 0.069–0.136, *p* < 0.001) and women (β = 0.054, 95% CI: 0.005–0.103, *p* = 0.031). As reported in Table 4, stratified by race/ethnicity, the positive relationship between metal objects and blood Cr was not observed in the Asian subgroup. Meanwhile, the positive relationship between metal objects and blood Co was not observed in the Asian and Hispanic subgroups.

**Fig. 2:**
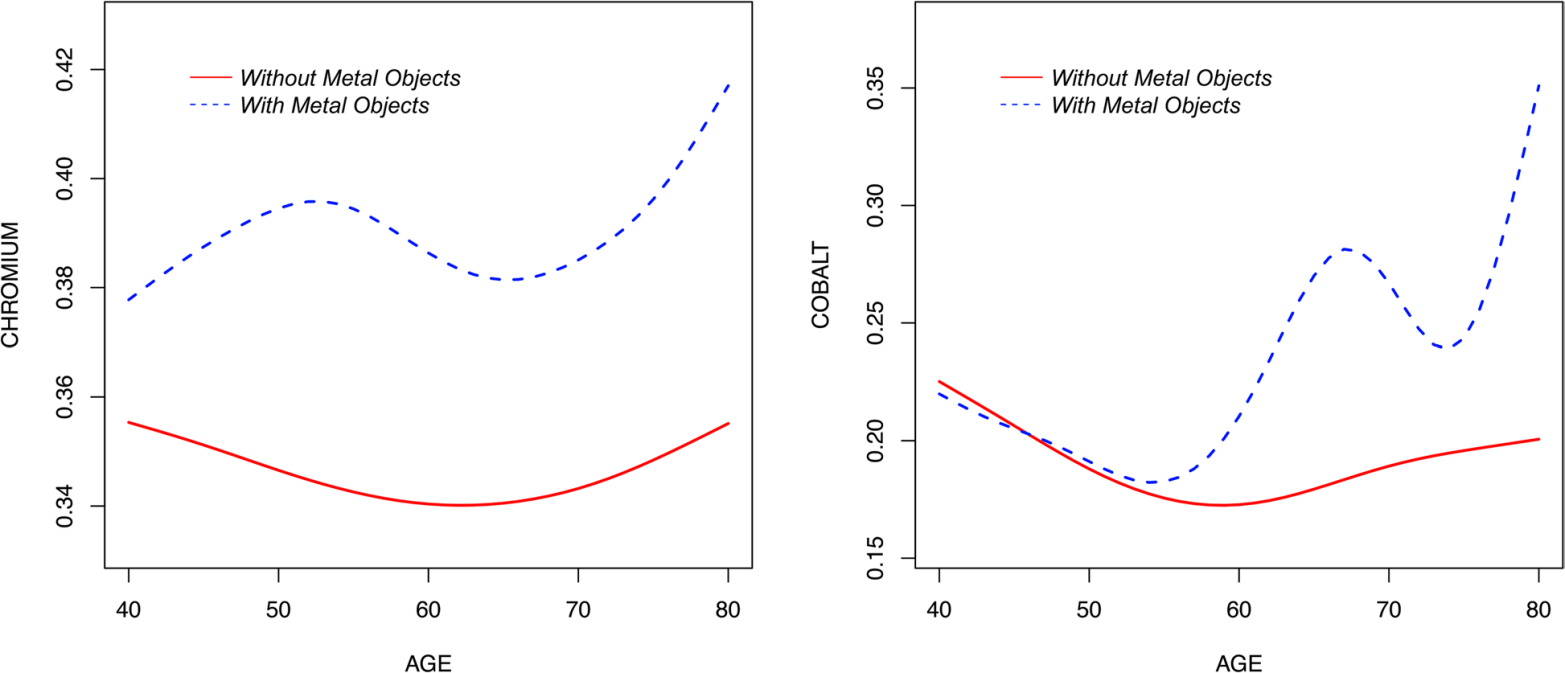
Smooth curve fittings between age and blood Cr (**a**) and Co (**b**) in different groups

Smooth curve fittings are shown in Fig. 2. Among participants with metal objects, a wave-shaped curve (Fig. 2a) was observed; senior citizens would have significantly higher blood Co (*p* = 0.008) but no association with blood Cr (*p* = 0.474). Among participants without metal objects, no association between age and blood Cr (*p* = 0.508) or Co (*p* = 0.269), although U-shaped curves (Fig. 2b) were observed.

## Discussion

This article used the NHANES database to explain the relationship between metal implants and concentrations of blood Cr and Co ions in a United States population surveyed between 2015 and 2018 through regression analysis, in an attempt to answer one of the most common questions doctors face with their patients: Doctor, will I have heavy metal poisoning from this implant? It is suggested that the presence of implant does cause the increase of blood ion concentration in the human body, but the concentration is far lower than the toxicological dose (Supplement Fig. 2). Based on Hart et al., [20] the acceptable upper limits of 2.56 μg/L for Cr and 2.02 μg/L for Co in whole blood have been proposed. However, based on different articles, the reference value varies. Another article [21] indicated that the concentration of Cr and Co in whole blood should be lower than 2.8 μg/L and 1.6 μg/L, respectively.

Prior to this, many studies focused on implant ion release. The release of ions in total hip arthroplasty mechanically assisted crevice corrosion is always a focus of attention [22–24]. However, most studies tend to focus on changes in metals such as titanium (Ti) and lead or other ions [25, 26]. Together, metal ion studies using the NHANES database are not primarily concerned with metal implants [27–29]. The traditional metal-on-metal hip implant contained the Co-Cr liner and the Ti shell. The modular junction between them (Co-Cr/Ti junction) has raised concerns over the corrosion potential at the dissimilar metal interface. Co-Cr/Ti junctions create more metal chips during wear and tear that can be absorbed by surrounding tissues, either deposited in the tissues or released into the blood [30–33].

Meanwhile, analysis of micronutrients in pregnant women revealed the accumulation of Co ions in pregnant women and analyzed its possible proportional relationship with vitamin B12 intake and smoking [34]. Tvermoes et al*.* and Chen et al*.* found that female adults had higher Co levels than male adults [35, 36]. However, this study showed that men were more likely than women to accumulate Co in the body. The controversy might be the difference between the studies with different confounders and follow-up times.

In addition, from the results of the stratified analysis, we can infer that there are racial differences in the accumulation of Cr in the body. Whites are the most likely to accumulate Cr, followed by African-American, followed by Hispanics, and Asian people are not easy to accumulate Cr. Similarly, Whites were most likely to accumulate Co, followed by African-American, and Asian and Hispanic people were not easy to accumulate Co. This paper combined the content of metal ions in the body with race analysis, and the mechanism may be related to the differences in genes related to metabolism between races, [37, 38] which requires further research. However, the lack of significance might due to restricted samples of Asians.

This study also noticed a distinct difference in the concentration of Cr and Co ions, which might be related to the distribution pattern of the two ions in tissues. It was found that Co was selectively leached from the alloy and released into the blood during plant corrosion. The free Cr released due to corrosion precipitated in local tissues as Cr phosphate, but did not form organometallic complexes in serum [39–43]. However, other studies have shown that Cr released from hip implants is preferentially distributed into serum, not red blood cells. Thus, the form of the Cr in the blood of these patients is in the non-toxic trivalent state, which is considered an essential nutrient [44]. At the same time, the serum metal ions can distinguish between patients with internal plant corrosion and patients without corrosion [45]. Taper junction corrosion and fretting, also known as mechanically assisted crevice corrosion, produces Co and Cr ions, fretting products, and corrosion debris, which may cause adverse local tissue reactions. And Cr and Co levels may help determine mechanically assisted crevice corrosion in a particular joint, and significantly elevated levels may explain symptoms, but not significantly elevated serum values [46–48].

What’s more, Co is part of vitamin B12, an indispensable heavy metal in the body. Studies have shown that Co can increase plasma high-density lipoprotein and decrease low-density lipoprotein, free fatty acids, and triglycerides in mice [48]. By regulating glycogen depot through suppressing glucagon signaling, Co could also influence body weight [49]. Di Santo et al*.* notes that although Cr and Co concentrations were elevated in patients with metal implants, they were far from the levels that would cause disease [50]. However, we should notice that the toxicity of a particular metal or metal ion could be different along the concentration of the metal or metal ion [51, 52].

This study has several shortcomings. First, as a cross-sectional study, temporal order and causality may not be clear. It is not possible to determine the sequential relationship between metal implants and blood ion levels in vivo. Second, due to privacy considerations, the NHANES database cannot provide the geographical location of participants, nor can it determine whether they live in an urban or rural environment, close to traffic or industrial areas, which makes it impossible to assess the influence of geographical environment and occupational exposure on metal ion content in the body. Meanwhile, the participants did not report the implantation site and the type of implants in each patient. This is a highly heterogenous group. There is no clinical specificity and no additional determination regarding the type of implant, number of implants, reason for the implants or duration that the implants have been present. Also, the implant might not contain Cr or Co, and the influence of weight-bearing joint and non-weight bearing joint implants might be different. However, this study did notice the elevated blood concentrations of Cr and Co in the participants with metal implants. Patients should be noticed the change after the operation. Although, the concentration is lower than the toxicological dose, it still needs longer follow-up.

**Table 1:** The characteristics of participants

	**Without Metal Objects**	**With Metal Objects**	**p**
Age (years)	56.6 ± 10.8	63.0 ± 11.4	<0.001
Sex			
Male	45.5%	50.0%	0.006
Female	54.5%	50.0%	
Race/Ethnicity			
Non-Hispanic White	63.3%	74.8%	<0.001
African American	11.8%	7.2%	
Non-Hispanic Asian	6.3%	2.7%	
Hispanic	14.1%	10.5%	
Others	4.6%	4.9%	
Body mass index (kg/m^2^)	30.1 ± 7.0	30.3 ± 6.3	0.245
Income to poverty ratio^a^			
<1	9.9%	8.6%	0.138
1-3	30.3%	29.5%	
>3	48.9%	52.3%	
Smoke≥100 cigarettes (%)	41.8%	52.0%	<0.001
Hemoglobin	14.2 ± 1.4	14.1 ± 1.4	0.814
Tap water^a^			
No	47.8%	45.4%	0.001
Yes	46.1%	50.7%	
Shellfish^a^			
No	43.2%	39.1%	<0.001
Yes	50.4%	56.5%	
Fish^a^			
No	26.1%	22.1%	<0.001
Yes	67.6%	73.6%	
Blood chromium	0.364 ± 0.358	0.438 ± 0.585	<0.001
Blood cobalt	0.180 ± 0.314	0.273 ± 0.650	<0.001

^a^variables with missing date as another category, the cumulation percent was not 100%

Mean±SD for continuous variables, *P* value was calculated by weighted linear regression model

% for categorical variables, *P* value was calculated by weighted chi-square test

**Table 2:** Association between metal objects and blood chromium/cobalt

	Unadjusted model	Minimally adjusted model	Fully adjusted model
Without metal objects	Reference	Reference	Reference
With metal objects			
Blood chromium			
β (95% CI)	0.074 (0.046, 0.102)	0.076 (0.047, 0.105)	0.072 (0.043, 0.102)
p	<0.001	<0.001	<0.001
Blood cobalt			
β (95% CI)	0.093 (0.064, 0.121)	0.083 (0.053, 0.113)	0.079 (0.049, 0.109)
p	<0.001	<0.001	<0.001

Unadjusted model: no covariates were adjusted

Minimally adjusted model: age, sex, body mass index, and race were adjusted

Fully adjusted model: age, sex, body mass index, race, data collection years, education level, marital status, income to poverty ratio, smoking behavior, body mass index, serum hemoglobin, tap water intake, shellfish intake, fish intake, tuna intake, and salmon intake were adjusted

**Table 3:** Association between metal objects and blood chromium/cobalt, stratified by sex

	Unadjusted model	Minimally adjusted model	Fully adjusted model
***Blood chromium***			
* Men*			
β (95% CI)	0.027 (-0.015, 0.070)	0.034 (-0.010, 0.078)	0.025 (-0.019, 0.070)
p	0.210	0.131	0.263
* Women*			
β (95% CI)	0.118 (0.081, 0.155)	0.114 (0.076, 0.153)	0.112 (0.074, 0.151)
p	<0.001	<0.001	<0.001
***Blood cobalt***			
* Men*			
β (95% CI)	0.123 (0.091, 0.155)	0.106 (0.073, 0.139)	0.102 (0.069, 0.136)
p	<0.001	<0.001	<0.001
* Women*			
β (95% CI)	0.067 (0.020, 0.113)	0.061 (0.012, 0.109)	0.054 (0.005, 0.103)
p	0.005	0.014	0.031

Unadjusted model: no covariates were adjusted

Minimally adjusted model: age, sex, body mass index, and race were adjusted

Fully adjusted model: age, sex, body mass index, race, data collection years, education level, marital status, income to poverty ratio, smoking behavior, body mass index, serum hemoglobin, tap water intake, shellfish intake, fish intake, tuna intake, and salmon intake were adjusted

**Table 4:** Association between metal objects and blood chromium/cobalt, stratified by race

	Unadjusted model	Minimally adjusted model	Fully adjusted model
***Blood chromium***			
* Non-Hispanic White*			
β (95% CI)	0.080 (0.026, 0.134)	0.094 (0.037, 0.150)	0.089 (0.033, 0.146)
p	0.004	0.001	0.002
* African American*			
β (95% CI)	0.043 (0.010, 0.077)	0.040 (0.006, 0.074)	0.038 (0.004, 0.073)
p	0.012	0.023	0.027
* Non-Hispanic Asian*			
β (95% CI)	0.022 (-0.015, 0.059)	0.021 (-0.016, 0.059)	0.027 (-0.012, 0.065)
p	0.240	0.264	0.175
* Hispanic*			
β (95% CI)	0.035 (0.004, 0.066)	0.034 (0.002, 0.065)	0.034 (0.002, 0.066)
p	0.029	0.035	0.035
***Blood cobalt***			
* Non-Hispanic White*			
β (95% CI)	0.124 (0.078, 0.171)	0.110 (0.061, 0.158)	0.104 (0.055, 0.153)
p	<0.001	<0.001	<0.001
* African American*			
β (95% CI)	0.039 (0.014, 0.064)	0.038 (0.013, 0.063)	0.037 (0.012, 0.063)
p	0.002	0.003	0.004
* Non-Hispanic Asian*			
β (95% CI)	0.007 (-0.020, 0.034)	0.016 (-0.011, 0.043)	0.019 (-0.007, 0.046)
p	0.593	0.240	0.152
* Hispanic*			
β (95% CI)	-0.025 (-0.120, 0.070)	-0.022 (-0.119, 0.075)	-0.018 (-0.115, 0.079)
p	0.609	0.654	0.718

Unadjusted model: no covariates were adjusted

Minimally adjusted model: age, sex, body mass index, and race were adjusted

Fully adjusted model: age, sex, body mass index, race, data collection years, education level, marital status, income to poverty ratio, smoking behavior, body mass index, serum hemoglobin, tap water intake, shellfish intake, fish intake, tuna intake, and salmon intake were adjusted

## Supplementary Information

**Additional file 1: Supplement Figure 1.** The directed acyclic graphs of confounders.

**Additional file 2: Supplement Figure 2.** The relationship between metal implants and concentrations of blood Cr/Co. It was made by the authors and approved by www.figdraw.com.

**Additional file 3: Supplement File 1.** R package codes.

**Additional file 4: Supplement Table 1.** Some other characteristics of participants

## Data Availability

The datasets used and/or analyzed during the current study are available from the corresponding author on reasonable request. The detailed information on the data is available at https://wwwn.cdc.gov/nchs/nhanes/.

## Abbreviations

NHANESL: National Health and Nutrition Examination Survey
BMI: Body mass index
Co: Cobalt
Cr: Chromium

## References

[1] Lohmander LS, Roos EM (2007). Clinical update: treating osteoarthritis. Lancet.

[2] Skou ST, Roos EM, Laursen MB, Rathleff MS, Arendt-Nielsen L, Simonsen O (2015). A Randomized, Controlled Trial of Total Knee Replacement. N Engl J Med.

[3] Bhandari M, Einhorn TA, Guyatt G, Schemitsch EH, Zura RD, Sprague S (2019). Total Hip Arthroplasty or Hemiarthroplasty for Hip Fracture. N Engl J Med.

[4] Chapman MW, Bowman WE, Csongradi JJ, Day LJ, Trafton PG, Bovill EG (1981). The use of Ender's pins in extracapsular fractures of the hip. J Bone Joint Surg Am.

[5] Rosen G, Murphy ML, Huvos AG, Gutierrez M, Marcove RC (1976). Chemotherapy, en bloc resection, and prosthetic bone replacement in the treatment of osteogenic sarcoma. Cancer..

[6] Stone GW, Maehara A, Ali ZA, Held C, Matsumura M, Kjøller-Hansen L (2020). Percutaneous Coronary Intervention for Vulnerable Coronary Atherosclerotic Plaque. J Am Coll Cardiol.

[7] Davis MA, Gilbert-Diamond D, Karagas MR, Li Z, Moore JH, Williams SM (2014). A dietary-wide association study (DWAS) of environmental metal exposure in US children and adults. PLoS ONE.

[8] Serdar MA, Akin BS, Razi C, Akin O, Tokgoz S, Kenar L (2012). The correlation between smoking status of family members and concentrations of toxic trace elements in the hair of children. Biol Trace Elem Res.

[9] Mishra S, Bharagava RN (2016). Toxic and genotoxic effects of hexavalent chromium in environment and its bioremediation strategies. J Environ Sci Health C Environ Carcinog Ecotoxicol Rev.

[10] Brodner W, Bitzan P, Meisinger V, Kaider A, Gottsauner-Wolf F, Kotz R (2003). Serum cobalt levels after metal-on-metal total hip arthroplasty. J Bone Joint Surg Am.

[11] Mathew SE, Xie Y, Bagheri L, Claton LE, Chu L, Badreldin A, et al. Are Serum Ion Levels Elevated in Pediatric Patients With Metal Implants? J Pediatr Orthop. 2022;42(3):162-8. 10.1097/BPO.0000000000001957 PMC8828674 34619722

[12] Jantzen C, Jørgensen HL, Duus BR, Sporring SL, Lauritzen JB (2013). Chromium and cobalt ion concentrations in blood and serum following various types of metal-on-metal hip arthroplasties: a literature overview. Acta Orthop.

[13] Bradberry SM, Wilkinson JM, Ferner RE (2014). Systemic toxicity related to metal hip prostheses. Clin Toxicol (Phila).

[14] Szczęsny G, Kopec M, Politis DJ, Kowalewski ZL, Łazarski A, Szolc T (2022). A review on biomaterials for orthopaedic surgery and traumatology: from past to present. Materials (Basel).

[15] Sampson B, Hart A (2012). Clinical usefulness of blood metal measurements to assess the failure of metal-on-metal hip implants. Ann Clin Biochem.

[16] Akbar M, Brewer JM, Grant MH (2011). Effect of chromium and cobalt ions on primary human lymphocytes in vitro. J Immunotoxicol.

[17] Wang K, Hu X, Li Z, Smolinski M, Xiao W, He J (2022). Association between snoring and insulin levels in the US population: a cross-sectional study. Sleep Breath.

[18] Rothrauff B, Tang Q, Wang J, He J (2022). Osteoarthritis is positively associated with self-reported sleep trouble in older adults. Aging Clin Exp Res.

[19] Norwood WP, Borgmann U, Dixon DG (2006). Saturation models of arsenic, cobalt, chromium and manganese bioaccumulation by Hyalella azteca. Environ Pollut.

[20] Hart AJ, Skinner JA, Winship P (2009). Circulating levels of cobalt and chromium from metal-on-metal hip replacement are associated with CD8+ T-cell lymphopenia. J Bone Joint Surg (Br).

[21] Iyengar GV (1987). Reference values for the concentrations of As, Cd Co, Cr, Cu, Fe, I, Hg, Mn, Mo, Ni, Pb, Se, and Zn in selected human tissues and body fluids. Biol Trace Elem Res.

[22] Garbuz DS, Tanzer M, Greidanus NV, Masri BA, Duncan CP (2010). The John Charnley Award: Metal-on-metal hip resurfacing versus large-diameter head metal-on-metal total hip arthroplasty: a randomized clinical trial. Clin Orthop Relat Res.

[23] Jacobs JJ, Cooper HJ, Urban RM, Wixson RL, Della Valle CJ (2014). What do we know about taper corrosion in total hip arthroplasty?. J Arthroplasty.

[24] Hussey DK, McGrory BJ (2017). Ten-Year Cross-Sectional Study of Mechanically Assisted Crevice Corrosion in 1352 Consecutive Patients With Metal-on-Polyethylene Total Hip Arthroplasty. J Arthroplasty.

[25] Mercuri LG, Miloro M, Skipor AK, Bijukumar D, Sukotjo C, Mathew MT (2018). Serum Metal Levels in Maxillofacial Reconstructive Surgery Patients: A Pilot Study. J Oral Maxillofac Surg.

[26] Tanoglu O, Say F, Yucens M, Alemdaroglu KB, Iltar S, Aydogan NH (2020). Titanium Alloy Intramedullary Nails and Plates Affect Serum Metal Ion Levels within the Fracture Healing Period. Biol Trace Elem Res.

[27] Madrigal JM, Persky V, Pappalardo A, Argos M (2018). Association of heavy metals with measures of pulmonary function in children and youth: Results from the National Health and Nutrition Examination Survey (NHANES). Environ Int.

[28] Padilla MA, Elobeid M, Ruden DM, Allison DB (2010). An examination of the association of selected toxic metals with total and central obesity indices: NHANES 99–02. Int J Environ Res Public Health.

[29] Wang X, Mukherjee B, Park SK. Associations of cumulative exposure to heavy metal mixtures with obesity and its comorbidities among U.S. adults in NHANES 2003–2014. Environ Int. 2018;121(Pt 1):683–94. 10.1016/j.envint.2018.09.035 PMC6268112 30316184

[30] Cooper HJ, Urban RM, Wixson RL, Meneghini RM, Jacobs JJ (2013). Adverse local tissue reaction arising from corrosion at the femoral neck-body junction in a dual-taper stem with a cobalt-chromium modular neck. J Bone Joint Surg Am.

[31] Di Laura A, Hothi HS, Henckel J, Kwon YM, Skinner JA, Hart AJ (2018). Retrieval Findings of Recalled Dual-Taper Hips. J Bone Joint Surg Am.

[32] Kim CH, Ryu JJ, Jeong MY, Kim JW, Chang JS, Yoon PW (2019). Serum Metal Ion Levels in Cementless Metal-On-Metal Total Hip Arthroplasty: Long-Term Follow-Up Trends. J Arthroplasty.

[33] Gkiatas I, Sharma AK, Greenberg A, Duncan ST, Chalmers BP, Sculco PK (2020). Serum metal ion levels in modular dual mobility acetabular components: A systematic review. J Orthop.

[34] Watson CV, Lewin M, Ragin-Wilson A, Jones R, Jarrett JM, Wallon K (2020). Characterization of trace elements exposure in pregnant women in the United States, NHANES 1999–2016. Environ Res.

[35] Tvermoes BE, Unice KM, Paustenbach DJ, Finley BL, Otani JM, Galbraith DA (2014). Effects and blood concentrations of cobalt after ingestion of 1 mg/d by human volunteers for 90 d. Am J Clin Nutr.

[36] Chen Y, Huang H, He X, Duan W, Mo X (2021). Sex differences in the link between blood cobalt concentrations and insulin resistance in adults without diabetes. Environ Health Prev Med.

[37] Meyer TE, Verwoert GC, Hwang SJ, CS, et al. Genome-wide association studies of serum magnesium, potassium, and sodium concentrations identify six Loci influencing serum magnesium levels. PLoS Genet. 2010;6(8):e1001045. 10.1371/journal.pgen.1001045 PMC2916845 20700443

[38] Stratakis N, Conti DV, Borras E (2020). Association of Fish Consumption and Mercury Exposure During Pregnancy With Metabolic Health and Inflammatory Biomarkers in Children. JAMA Netw Open.

[39] Shao W, Liu Q, He X, Liu H, Gu A, Jiang Z (2017). Association between level of urinary trace heavy metals and obesity among children aged 6–19 years: NHANES 1999–2011. Environ Sci Pollut Res Int.

[40] Jin R, Zhu X, Shrubsole MJ, Yu C, Xia Z, Dai Q (2018). Associations of renal function with urinary excretion of metals: Evidence from NHANES 2003–2012. Environ Int.

[41] Martin JR, Camp CL, Wyles CC, Taunton MJ, Trousdale RT, Lewallen DG (2016). Increased Femoral Head Offset is Associated With Elevated Metal Ions in Asymptomatic Patients With Metal-on-Polyethylene Total Hip Arthroplasty. J Arthroplasty.

[42] Maurer-Ertl W, Pranckh-Matzke D, Friesenbichler J, Bratschitsch G, Holzer LA, Maier M (2017). Clinical Results and Serum Metal Ion Concentrations following Ceramic-on-Metal Total Hip Arthroplasty at a Mean Follow-Up of 60 Months. Biomed Res Int.

[43] Gilbert JL, Buckley CA, Jacobs JJ. In vivo corrosion of modular hip prosthesis components in mixed and similar metal combinations. The effect of crevice, stress, motion, and alloy coupling. J Biomed Mater Res. 1993;27(12):1533–44. 10.1002/jbm.820271210 8113241

[44] Ho JH, Leikin JB, Dargan PI, Archer JRH, Wood DM, Brent J (2017). Metal-on-Metal Hip Joint Prostheses: a Retrospective Case Series Investigating the Association of Systemic Toxicity with Serum Cobalt and Chromium Concentrations. J Med Toxicol.

[45] Klasan A, Meine E, Fuchs-Winkelmann S, Efe T, Boettner F, Heyse TJ (2019). Are Serum Metal Ion Levels a Concern at Mid-term Followup of Revision Knee Arthroplasty With a Metal-on-metal Hinge Design?. Clin Orthop Relat Res.

[46] Zuiderbaan HA, Visser D, Sierevelt IN, Penders J, Verhart J, Vergroesen DA. Long-term clinical results of the Metasul metal-on-metal total hip arthroplasty: 12.6 years follow-up of 128 primary total hip replacements. Hip Int. 2018;28(3):330–5. 10.5301/hipint.5000574 29048689

[47] McGrory BJ, Payson AM, MacKenzie JA (2017). Elevated Intra-Articular Cobalt and Chromium Levels in Mechanically Assisted Crevice Corrosion in Metal-on-Polyethylene Total Hip Arthroplasty. J Arthroplasty.

[48] Kawakami T, Hanao N, Nishiyama K, Kadota Y, Inoue M, Sato M (2012). Differential effects of cobalt and mercury on lipid metabolism in the white adipose tissue of high-fat diet-induced obesity mice. Toxicol Appl Pharmacol.

[49] Tascilar ME, Ozgen IT, Abaci A, Serdar M, Aykut O (2011). Trace elements in obese Turkish children. Biol Trace Elem Res.

[50] Di Santo P, Motazedian P, Jung RG, Simard T, Ramirez FD, Chong AY (2018). Evaluation of Cobalt and Chromium Levels Following Implantation of Cobalt Chromium Coronary Stents: A Pilot Study. Heart Lung Circ.

[51] Bauer C, Stotter C, Jeyakumar V, Niculescu-Morzsa E, Simlinger B, Rodríguez Ripoll M, Klestil T, Franek F, Nehrer S. Concentration-Dependent Effects of Cobalt and Chromium Ions on Osteoarthritic Chondrocytes. Cartilage. 2021;13(2_suppl):908S-919S. 10.1177/1947603519889389 PMC8721608 31779468

[52] Chen Z, Zhang Z, Qi J, You J, Ma J, Chen L (2023). Colorimetric detection of heavy metal ions with various chromogenic materials: Strategies and applications. J Hazard Mater.

