# Supplementary figures and images for: Accumulation of blood chromium and cobalt in the participants with metal objects: findings from the 2015 to 2018 National Health and Nutrition Examination Survey (NHANES)
Source: BMC Geriatr. 2023 Feb 3;23:72. doi: 10.1186/s12877-022-03710-3 (PMC9898935; doi:10.1186/s12877-022-03710-3)

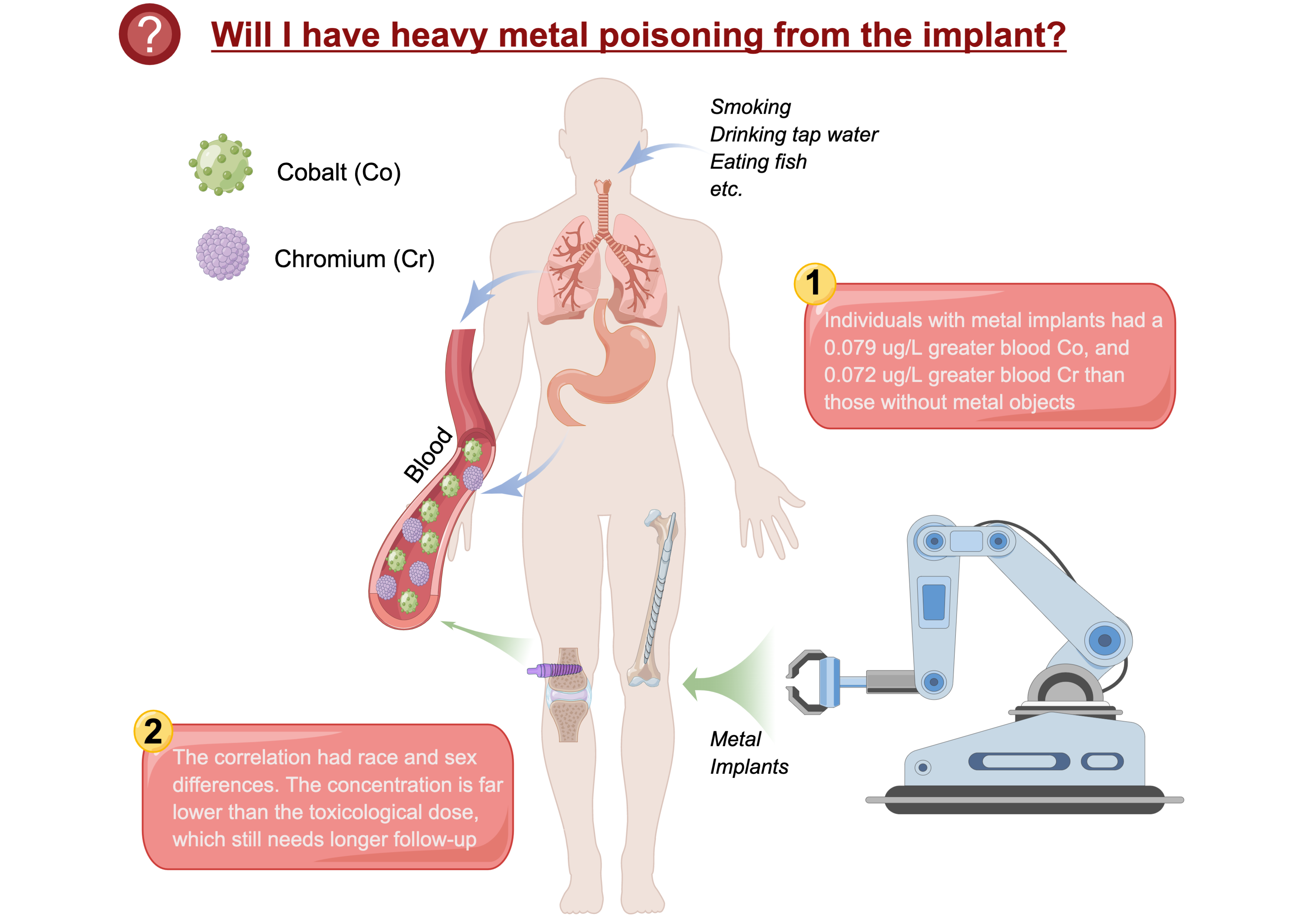

Supplement: Supplementary file 2 — Additional file 2: Supplement Figure 2. The relationship between metal implants and concentrations of blood Cr/Co. It was made by the authors and approved by www.figdraw.com. [file 12877_2022_3710_MOESM2_ESM.png]
