# Supplementary material for: Accumulation of blood chromium and cobalt in the participants with metal objects: findings from the 2015 to 2018 National Health and Nutrition Examination Survey (NHANES)
Source: BMC Geriatr. 2023 Feb 3;23:72. doi: 10.1186/s12877-022-03710-3 (PMC9898935; doi:10.1186/s12877-022-03710-3)
Supplement: Supplementary file 3 — Additional file 3: Supplement File 1. R package codes. [file 12877_2022_3710_MOESM3_ESM.docx]

R version 3.4.3 (2017-11-30) -- "Kite-Eating Tree"

Copyright (C) 2017 The R Foundation for Statistical Computing

Platform: x86_64-w64-mingw32/x64 (64-bit)

R «◊‘”…»Ìº˛£¨≤ª¥¯»Œ∫Œµ£±£°£

‘⁄ƒ≥–©Ãıº˛œ¬ƒ„ø…“‘Ω´∆‰◊‘”……¢≤º°£

”√'license()'ªÚ'licence()'¿¥ø¥…¢≤ºµƒœÍœ∏Ãıº˛°£

R «∏ˆ∫œ◊˜º∆ªÆ£¨”––Ì∂‡»ÀŒ™÷Æ◊ˆ≥ˆ¡Àπ±œ◊.

”√'contributors()'¿¥ø¥∫œ◊˜’ﬂµƒœÍœ∏«Èøˆ

”√'citation()'ª·∏ÊÀﬂƒ„»Á∫Œ‘⁄≥ˆ∞ÊŒÔ÷–’˝»∑µÿ“˝”√RªÚR≥Ã–Ú∞¸°£

”√'demo()'¿¥ø¥“ª–© æ∑∂≥Ã–Ú£¨”√'help()'¿¥‘ƒ∂¡‘⁄œﬂ∞Ô÷˙Œƒº˛£¨ªÚ

”√'help.start()'Õ®π˝HTML‰Ø¿¿∆˜¿¥ø¥∞Ô÷˙Œƒº˛°£

”√'q()'ÕÀ≥ˆR.

> R.Version4RUN<-343;

> R.LibLocation <- "C:/Users/tangqi/AppData/Roaming/EmpowerRCH/R343/library"

> #***************** Regarding ALL Following R Functions ********************

> #***** COPYRIGHT (c) 2010 X&Y Solutions, ALL RIGHT RESERVED ***************

> #******************* www.EmpowerStats.com *********************************

> #**************************************************************************

> Sys.setlocale("LC_TIME", "C")

[1] "C"

> library(doBy,lib.loc=R.LibLocation)

Warning message:

≥Ãº≠∞¸'doBy' «”√R∞Ê±æ3.4.4 ¿¥Ω®‘Ïµƒ

> library(plotrix,lib.loc=R.LibLocation)

Warning message:

≥Ãº≠∞¸'plotrix' «”√R∞Ê±æ3.4.4 ¿¥Ω®‘Ïµƒ

> library(stringi,lib.loc=R.LibLocation)

Warning message:

≥Ãº≠∞¸'stringi' «”√R∞Ê±æ3.4.4 ¿¥Ω®‘Ïµƒ

> library(stringr,lib.loc=R.LibLocation)

Warning message:

≥Ãº≠∞¸'stringr' «”√R∞Ê±æ3.4.4 ¿¥Ω®‘Ïµƒ

> library(survival,lib.loc=R.LibLocation)

> library(rms,lib.loc=R.LibLocation)

‘ÿ»Î–Ë“™µƒ≥Ãº≠∞¸£∫Hmisc

‘ÿ»Î–Ë“™µƒ≥Ãº≠∞¸£∫lattice

‘ÿ»Î–Ë“™µƒ≥Ãº≠∞¸£∫Formula

‘ÿ»Î–Ë“™µƒ≥Ãº≠∞¸£∫ggplot2

‘ÿ»Î≥Ãº≠∞¸£∫'Hmisc'

The following objects are masked from 'package:base':

format.pval, units

‘ÿ»Î–Ë“™µƒ≥Ãº≠∞¸£∫SparseM

‘ÿ»Î≥Ãº≠∞¸£∫'SparseM'

The following object is masked from 'package:base':

backsolve

Warning messages:

1: ≥Ãº≠∞¸'rms' «”√R∞Ê±æ3.4.4 ¿¥Ω®‘Ïµƒ

2: ≥Ãº≠∞¸'Hmisc' «”√R∞Ê±æ3.4.4 ¿¥Ω®‘Ïµƒ

3: ≥Ãº≠∞¸'Formula' «”√R∞Ê±æ3.4.4 ¿¥Ω®‘Ïµƒ

4: ≥Ãº≠∞¸'ggplot2' «”√R∞Ê±æ3.4.4 ¿¥Ω®‘Ïµƒ

5: ≥Ãº≠∞¸'SparseM' «”√R∞Ê±æ3.4.4 ¿¥Ω®‘Ïµƒ

> library(nnet,lib.loc=R.LibLocation)

> library(car,lib.loc=R.LibLocation)

‘ÿ»Î–Ë“™µƒ≥Ãº≠∞¸£∫carData

‘ÿ»Î≥Ãº≠∞¸£∫'car'

The following objects are masked from 'package:rms':

Predict, vif

Warning messages:

1: ≥Ãº≠∞¸'car' «”√R∞Ê±æ3.4.4 ¿¥Ω®‘Ïµƒ

2: ≥Ãº≠∞¸'carData' «”√R∞Ê±æ3.4.4 ¿¥Ω®‘Ïµƒ

> library(mgcv,lib.loc=R.LibLocation)

‘ÿ»Î–Ë“™µƒ≥Ãº≠∞¸£∫nlme

This is mgcv 1.8-22. For overview type 'help("mgcv-package")'.

‘ÿ»Î≥Ãº≠∞¸£∫'mgcv'

The following object is masked from 'package:nnet':

multinom

> pdfwd<-6; pdfht<-6

> setwd("C:/Users/tangqi/Desktop/PROJ1_4_tbl")

> load("C:/Users/tangqi/Desktop/metal20211115.Rdata")

> if (length(which(ls()=="EmpowerStatsR"))==0) EmpowerStatsR<-get(ls()[1])

> names(EmpowerStatsR)<-toupper(names(EmpowerStatsR))

> #--#

>

> vname<-c("_N_","_STAT_","_TOTAL_","SEQN","METAL_OBJECTS","METAL_OBJECTS.0","METAL_OBJECTS.1")

> vlabel<-c("—˘±æ¡ø(%)","Õ≥º∆¡ø","∫œº∆","SEQN","METAL_OBJECTS"," 0"," 1")

> vname<-c(vname,"CHROMIUM","COBALT","MERCURY","SELENIUM","MANGANESE")

> vlabel<-c(vlabel,"CHROMIUM","COBALT","MERCURY","SELENIUM","MANGANESE")

> vname<-c(vname,"LEAD","CADMIUM","CYCLE","CYCLE.9","CYCLE.10")

> vlabel<-c(vlabel,"LEAD","CADMIUM","CYCLE"," 9"," 10")

> vname<-c(vname,"SEX","SEX.1","SEX.2","AGE","RACE","RACE.1","RACE.2","RACE.3","RACE.4","RACE.5")

> vlabel<-c(vlabel,"SEX"," 1"," 2","AGE","RACE"," 1"," 2"," 3"," 4"," 5")

> vname<-c(vname,"EDUCATION","EDUCATION.1","EDUCATION.2","EDUCATION.3")

> vlabel<-c(vlabel,"EDUCATION"," 1"," 2"," 3")

> vname<-c(vname,"MARITAL","MARITAL.1","MARITAL.2","WEIGHT_QUAN")

> vlabel<-c(vlabel,"MARITAL"," 1"," 2","WEIGHT_QUAN")

> vname<-c(vname,"INCOME","INCOME.1","INCOME.2","INCOME.3","INCOME.4")

> vlabel<-c(vlabel,"INCOME"," 1"," 2"," 3"," 4")

> vname<-c(vname,"WEIGHT","HEIGHT","BMI","WAIST","HEMOGLOBIN")

> vlabel<-c(vlabel,"WEIGHT","HEIGHT","BMI","WAIST","HEMOGLOBIN")

> vname<-c(vname,"TAP_WATER","TAP_WATER.0","TAP_WATER.1","TAP_WATER.2")

> vlabel<-c(vlabel,"TAP_WATER"," 0"," 1"," 2")

> vname<-c(vname,"SHELLFISH","SHELLFISH.0","SHELLFISH.1","SHELLFISH.2")

> vlabel<-c(vlabel,"SHELLFISH"," 0"," 1"," 2")

> vname<-c(vname,"FISH","FISH.0","FISH.1","FISH.2","TUNA","TUNA.0","TUNA.1","TUNA.2")

> vlabel<-c(vlabel,"FISH"," 0"," 1"," 2","TUNA"," 0"," 1"," 2")

> vname<-c(vname,"SALMON","SALMON.0","SALMON.1","SALMON.2")

> vlabel<-c(vlabel,"SALMON"," 0"," 1"," 2")

> vname<-c(vname,"SMOKE","SMOKE.0","SMOKE.1")

> vlabel<-c(vlabel,"SMOKE"," 0"," 1")

> slt.vname<-c()

>

> library(mgcv,lib.loc=R.LibLocation)

> library(gdata,lib.loc=R.LibLocation)

gdata: Unable to locate valid perl interpreter

gdata:

gdata: read.xls() will be unable to read Excel XLS and XLSX files

gdata: unless the 'perl=' argument is used to specify the location of a

gdata: valid perl intrpreter.

gdata:

gdata: (To avoid display of this message in the future, please ensure

gdata: perl is installed and available on the executable search path.)

gdata: Unable to load perl libaries needed by read.xls()

gdata: to support 'XLX' (Excel 97-2004) files.

gdata: Unable to load perl libaries needed by read.xls()

gdata: to support 'XLSX' (Excel 2007+) files.

gdata: Run the function 'installXLSXsupport()'

gdata: to automatically download and install the perl

gdata: libaries needed to support Excel XLS and XLSX formats.

‘ÿ»Î≥Ãº≠∞¸£∫'gdata'

The following object is masked from 'package:stats':

nobs

The following object is masked from 'package:utils':

object.size

The following object is masked from 'package:base':

startsWith

Warning message:

≥Ãº≠∞¸'gdata' «”√R∞Ê±æ3.4.4 ¿¥Ω®‘Ïµƒ

>

> ofname<-"PROJ1_4_tbl";

> WD<-EmpowerStatsR; wd.subset="";

> svy.DSN.YN <- FALSE;

> weights<-WD$WEIGHT_QUAN;weights.var <- 'weight_quan';

> WD<-cbind(WD,weights); WD<-WD[!is.na(weights),];

> title<-"∆Ωª¨«˙œﬂƒ‚∫œ";

> attach(WD)

The following object is masked _by_ .GlobalEnv:

weights

> subjvname<-NA;

> yv<-cbind(CHROMIUM,COBALT);

> yvname<-c('CHROMIUM','COBALT');

> yvar<-c('CHROMIUM','COBALT');

> ydist<-c('gaussian','gaussian');

> ylink<-c('identity','identity');

> ylv<-c(0,0);

> xv<-cbind(RACE,EDUCATION,MARITAL,WEIGHT_QUAN,INCOME,WEIGHT,HEIGHT,BMI,WAIST,HEMOGLOBIN,TAP_WATER,SHELLFISH,FISH,TUNA,SALMON,SMOKE);

> xvname<-c('RACE','EDUCATION','MARITAL','WEIGHT_QUAN','INCOME','WEIGHT','HEIGHT','BMI','WAIST','HEMOGLOBIN','TAP_WATER','SHELLFISH','FISH','TUNA','SALMON','SMOKE');

> xvar<-c('RACE','EDUCATION','MARITAL','WEIGHT_QUAN','INCOME','WEIGHT','HEIGHT','BMI','WAIST','HEMOGLOBIN','TAP_WATER','SHELLFISH','FISH','TUNA','SALMON','SMOKE');

> xlv<-c(5,3,2,0,4,0,0,0,0,0,3,3,3,3,3,2);

> sxf<-c(NA,0,0,0,0,0,0,0,0,0,0,0,0,0,0,0,0)[-1];

> sv<-cbind(AGE);

> svname<-c('AGE');

> svar<-c('AGE');

> sdf<-c(NA,0)[-1];

> slv<-c(0);

> av<-NA; avname<-NA; avlbl<-NA; nadj<-0; alv<-NA;

> timev<-NA; timevname<-NA;

> bv<-NA; bvar<-NA;

> colv<-NA; colvname<-NA;

> v.start<-NA; vname.start<-NA;

> v.stop<-NA; vname.stop<-NA;

> par1<-1;dec<-4;parm<-c(NA, NA, NA, NA, 0);

> if (!exists("pdfwd")) pdfwd<-6;

> if (!exists("pdfht")) pdfht<-6;

> ##R package## mgcv gdata ##R package##;

> vec2shift<-function(vnew,vorg,f, opt) {

+ if (is.na(f[1])) {

+ mean1<-mean(vorg)

+ if (opt=="logit") mean1<-log(mean1/(1-mean1))

+ if (opt=="log") mean1<-log(mean1)

+ vnew<-vnew+(mean1-mean(vnew))

+ } else {

+ mean1<-tapply(vorg,factor(f),mean)

+ if (opt=="logit") mean1<-log(mean1/(1-mean1))

+ if (opt=="log") mean1<-log(mean1)

+ mean2<-tapply(vnew,factor(f),mean); meand<-mean1-mean2; lvf<-levels(factor(f))

+ for (z in (1:length(lvf))) {vnew[factor(f)==lvf[z]]<-vnew[factor(f)==lvf[z]]+meand[z]; }

+ }

+ return(vnew)

+ }

> getNumber<-function(str, n) {

+ str<-substr(str,2,nchar(str)-1)

+ for (i in (1:nchar(str))) {if (substr(str,i,i)==",") {p=i; break}; }

+ ifelse(n==1,return(substr(str,1,p-1)),return(substr(str,p+1,nchar(str))))

+ }

> legLocate<-function(x,y) {

+ x[is.infinite(y)]<-NA

+ y[is.infinite(y)]<-NA

+ xmin<-min(x,na.rm=TRUE); xmax<-max(x,na.rm=TRUE)

+ ymin<-min(y,na.rm=TRUE); ymax<-max(y,na.rm=TRUE)

+ yoff<-(ymax-ymin); tmp<-table(cut(x,3),cut(y,4))

+ tmp.r=which.min(tmp[,4]);tmp.c=4

+ if (tmp[2,1]==0) {tmp.r=2;tmp.c=1}

+ if (tmp[1,1]==0) {tmp.r=1;tmp.c=1}

+ if (tmp[3,1]==0) {tmp.r=3;tmp.c=1}

+ if (tmp[2,4]==0) {tmp.r=2;tmp.c=4}

+ if (tmp[1,4]==0) {tmp.r=1;tmp.c=4}

+ if (tmp[3,4]==0) {tmp.r=3;tmp.c=4}

+ pos.y<-colnames(tmp)[tmp.c]; pos.x<-rownames(tmp)[tmp.r]; pct<-0.15

+ if (tmp.c==4) {

+ if (min(tmp[,4])>0) {pct<-0.3}

+ ymax<-ymax+yoff*pct; legy<-ymax;ymin<-ymin-yoff*0.1

+ }

+ if (tmp.c==1) {

+ if (min(tmp[,1])>0) {pct<-0.3}

+ legy<-as.numeric(getNumber(pos.y,2));ymin<-ymin-yoff*pct;ymax=ymax+yoff*0.1

+ }

+ legx<-as.numeric(getNumber(pos.x,1))

+ return(cbind(xmin,xmax,ymin,ymax,legx,legy))

+ }

> mat2htmltable<-function(mat) {

+ t1<- apply(mat,1,function(z) paste(z,collapse="</td><td>"))

+ t2<- paste("<tr><td>",t1,"</td></tr>")

+ return(paste(t2,collapse=" "))

+ }

> setgam<-function(fml,yi) {

+ if (ydist[yi]=="") ydist[yi]<-"gaussian"

+ if (ydist[yi]=="exact") ydist[yi]<-"binomial"

+ if (ydist[yi]=="breslow") ydist[yi]<-"binomial"

+ if (ydist[yi]=="gaussian") mdl<-gam(formula(fml),weights=wd$weights,data=wd, family=gaussian(link="identity"))

+ if (ydist[yi]=="binomial") mdl<-gam(formula(fml),weights=wd$weights,data=wd, family=binomial(link="logit"))

+ if (ydist[yi]=="poisson") mdl<-gam(formula(fml),weights=wd$weights,data=wd, family=poisson(link="log"))

+ if (ydist[yi]=="gamma") mdl<-gam(formula(fml),weights=wd$weights,data=wd, family=Gamma(link="inverse"))

+ if (ydist[yi]=="negbin") mdl<-gam(formula(fml),weights=wd$weights,data=wd, family=negbin(c(1,10), link="log"))

+ return(mdl)

+ }

> gam2htmltable<-function(mdl) {

+ gs<-summary(mdl)

+ np<-length(gs$p.coeff)

+ coe<-gs$p.table

+ if (gs$family[[2]]=="log" | gs$family[[2]]=="logit") {

+ cnames<-c(colnames(coe),"exp(est)","95%CI low","95%CI upp")

+ coe<- cbind(coe, exp(coe[,1]), exp(coe[,1]-1.96*coe[,2]), exp(coe[,1]+1.96*coe[,2]))

+ }

+ if (gs$family[[2]]=="identity") {

+ cnames<-c(colnames(coe),"95%CI low","95%CI upp")

+ coe<- cbind(coe, coe[,1]-1.96*coe[,2], coe[,1]+1.96*coe[,2])

+ }

+ oo1<-cbind(c("",rownames(coe)),rbind(cnames,round(coe,dec)))

+ oo<-c("</br>Linear terms effect<table border=3>",mat2htmltable(oo1),"</table>")

+ if (!is.null(gs$pTerms.table)) {

+ xsq<-gs$pTerms.table

+ oo2<-cbind(c("",rownames(xsq)),rbind(colnames(xsq),round(xsq,dec)))

+ oo<-c(oo, "</br>Chi-square tests for linear terms<table border=3>",mat2htmltable(oo2),"</table>")

+ }

+ if (!is.null(gs$s.table)) {

+ stb<-gs$s.table

+ oo3<-cbind(c("",rownames(stb)),rbind(colnames(stb),round(stb,dec)))

+ oo<-c(oo, "</br>Approximate significance of smooth terms<table border=3>",mat2htmltable(oo3),"</table>")

+ }

+ p0<-c("N:", gs$n)

+ p1<-c("Adj. r-square:", round(gs$r.sq,4))

+ p2<-c("Deviance explained:", round(gs$dev.expl,4))

+ p3<-c("UBRE score (sp.criterion):", round(gs$sp.criterion,4))

+ p4<-c("Scale estimate:", gs$scale)

+ p5<-c("family:", gs$family[[1]])

+ p6<-c("link function:", gs$family[[2]])

+ oo4<-rbind(p0,p1,p2,p3,p4,p5,p6)

+ oo<-c(oo, "</br>Model statistics<table border=3>",mat2htmltable(oo4),"</table>")

+ return(oo)

+ }

> gam2pngs<-function(mdl,yi,xi) {

+ pred<-predict.gam(mdl,type="terms",se.fit=TRUE)

+ mfit<-NA; sfit<-NA; tmp.cname<-NA; kk0<-NA

+ if (xi==0) {kb=1; ke=ns;} else {kb=xi; ke=xi;}

+ for (k in (kb:ke)) {

+ if (slv[k]==0) {

+ mfit<-cbind(mfit,apply(cbind(0,pred$fit[,sxterms[k,]]),1,sum));

+ sfit<-cbind(sfit,apply(cbind(0,pred$se.fit[,sxterms[k,]]),1,sum));

+ tmp.cname<-c(tmp.cname,svname[k])

+ kk0<-c(kk0,k)

+ }

+ }

+ tmp.cname<-tmp.cname[-1]; kk0<-kk0[-1]

+ mfit<-matrix(mfit[,-1],ncol=length(tmp.cname)); colnames(mfit)<-paste(tmp.cname,".fit",sep="");

+ sfit<-matrix(sfit[,-1],ncol=length(tmp.cname)); colnames(sfit)<-paste(tmp.cname,".se",sep="");

+ if (!is.na(colvname)) {tmpfac<-wd[,colvname];} else {tmpfac<-NA;}

+ if (mdl$family[2]=="logit") {

+ mfit<-apply(mfit,2,function(z) vec2shift(z,mdl$fitted.value,tmpfac,"logit"))

+ mfit.low<-mfit-1.96*sfit; mfit.low<-matrix(exp(mfit.low)/(1+exp(mfit.low)),ncol=length(tmp.cname))

+ mfit.upp<-mfit+1.96*sfit; mfit.upp<-matrix(exp(mfit.upp)/(1+exp(mfit.upp)),ncol=length(tmp.cname))

+ mfit<-matrix(exp(mfit)/(1+exp(mfit)),ncol=length(tmp.cname))

+ colnames(mfit.low)<-paste(tmp.cname,".low",sep="");

+ colnames(mfit.upp)<-paste(tmp.cname,".upp",sep="");

+ colnames(mfit)<-paste(tmp.cname,".fit",sep="");

+ ww<-cbind(wd,mfit); if (is.na(colvname)) ww<-cbind(ww,mfit.low,mfit.upp)

+ } else if (mdl$family[2]=="log") {

+ mfit<-apply(mfit,2,function(z) vec2shift(z,mdl$fitted.value,tmpfac,"log"))

+ mfit.low<-mfit-1.96*sfit; mfit.low<-matrix(exp(mfit.low),ncol=length(tmp.cname))

+ mfit.upp<-mfit+1.96*sfit; mfit.upp<-matrix(exp(mfit.upp),ncol=length(tmp.cname))

+ mfit<-matrix(exp(mfit),ncol=length(tmp.cname))

+ colnames(mfit.low)<-paste(tmp.cname,".low",sep="");

+ colnames(mfit.upp)<-paste(tmp.cname,".upp",sep="");

+ colnames(mfit)<-paste(tmp.cname,".fit",sep="");

+ ww<-cbind(wd,mfit); if (is.na(colvname)) ww<-cbind(ww,mfit.low,mfit.upp)

+ } else if (mdl$family[2]=="identity") {

+ mfit<-apply(mfit,2,function(z) vec2shift(z,mdl$fitted.value,tmpfac," "))

+ ww<-cbind(wd,mfit,sfit)

+ } else {

+ ww<-cbind(wd,mfit,sfit)

+ }

+ if (xi!=0) {xf<-paste(ofname,yvar[yi],svar[xi],"gam.xls",sep="_");} else {xf<-paste(ofname,yvar[yi], "gam.xls", sep="_");}

+ write.table(ww,file=xf,row.names=FALSE,col.names=TRUE,sep="\t",append=FALSE,quote=FALSE)

+ px<-c(20,1:9); gg<-"";

+ for (k in kk0) {

+ cname1<-paste(svname[k],".fit",sep=""); y.tmp<-mfit[,cname1]

+ if (mdl$family[2]=="logit" | mdl$family[2]=="log") {

+ cname2<-paste(svname[k],".low",sep=""); y.low<-mfit.low[,cname2]

+ cname3<-paste(svname[k],".upp",sep=""); y.upp<-mfit.upp[,cname3]

+ } else {

+ cname2<-paste(svname[k],".se",sep=""); se.tmp<-sfit[,cname2];

+ y.low<-y.tmp-1.96*se.tmp; y.upp<-y.tmp+1.96*se.tmp

+ }

+ x.tmp<-wd[,svname[k]];

+ pngf<-paste(ofname,yvar[yi],svar[k],"smooth.png",sep="_")

+ pdff<-paste(ofname,yvar[yi],svar[k],"smooth.pdf",sep="_")

+ pngf0<-paste(ofname,yvar[yi],svar[k],"smooth1.png",sep="_")

+ pdff0<-paste(ofname,yvar[yi],svar[k],"smooth1.pdf",sep="_")

+ if (is.na(colvname)) {

+ if (is.na(parm[1])) {tmp.col<-c("red","blue");} else {tmp.col<-rep("black",2);}

+ xy<-legLocate(c(x.tmp,x.tmp),c(y.low,y.upp))

+ png(pngf,width=720,height=560)

+ plot(y.tmp~x.tmp,ylim=c(xy[3],xy[4]),xlim=c(xy[1],xy[2]),col=tmp.col[1],type="p", pch=20, ylab="", xlab="")

+ par(new=TRUE);

+ plot(y.low~x.tmp,ylim=c(xy[3],xy[4]),xlim=c(xy[1],xy[2]),col=tmp.col[2], type="p", pch=1, ylab="", xlab="")

+ par(new=TRUE);

+ plot(y.upp~x.tmp,ylim=c(xy[3],xy[4]),xlim=c(xy[1],xy[2]),col=tmp.col[2], type="p", pch=1, ylab=yb[yi], xlab=sb[k])

+ dev.off()

+

+ pdf(pdff,width=pdfwd, height=pdfht, family="Helvetica");

+ plot(y.tmp~x.tmp,ylim=c(xy[3],xy[4]),xlim=c(xy[1],xy[2]),col=tmp.col[1],type="p", pch=20, ylab="", xlab="")

+ par(new=TRUE);

+ plot(y.low~x.tmp,ylim=c(xy[3],xy[4]),xlim=c(xy[1],xy[2]),col=tmp.col[2], type="p", pch=1, ylab="", xlab="")

+ par(new=TRUE);

+ plot(y.upp~x.tmp,ylim=c(xy[3],xy[4]),xlim=c(xy[1],xy[2]),col=tmp.col[2], type="p", pch=1, ylab=yb[yi], xlab=sb[k])

+ dev.off()

+

+ png(pngf0,width=720,height=560)

+ tmp.ord<-order(x.tmp); x.tmp0<-x.tmp[tmp.ord];

+ y.tmp0<-y.tmp[tmp.ord];y.low0<-y.low[tmp.ord];y.upp0<-y.upp[tmp.ord]

+ plot(y.tmp0~x.tmp0,ylim=c(xy[3],xy[4]),xlim=c(xy[1],xy[2]),col=tmp.col[1],type="l", lty=1, lwd=2, ylab="", xlab="")

+ par(new=TRUE);

+ plot(y.low0~x.tmp0,ylim=c(xy[3],xy[4]),xlim=c(xy[1],xy[2]),col=tmp.col[2], type="l", lty=3, lwd=1, ylab="", xlab="")

+ par(new=TRUE);

+ plot(y.upp0~x.tmp0,ylim=c(xy[3],xy[4]),xlim=c(xy[1],xy[2]),col=tmp.col[2], type="l", lty=3, lwd=1, ylab=yb[yi], xlab=sb[k])

+ rug(x.tmp0)

+ dev.off()

+

+ pdf(pdff0,width=pdfwd, height=pdfht, family="Helvetica");

+ tmp.ord<-order(x.tmp); x.tmp0<-x.tmp[tmp.ord];

+ y.tmp0<-y.tmp[tmp.ord];y.low0<-y.low[tmp.ord];y.upp0<-y.upp[tmp.ord]

+ plot(y.tmp0~x.tmp0,ylim=c(xy[3],xy[4]),xlim=c(xy[1],xy[2]),col=tmp.col[1],type="l", lty=1, lwd=2, ylab="", xlab="")

+ par(new=TRUE);

+ plot(y.low0~x.tmp0,ylim=c(xy[3],xy[4]),xlim=c(xy[1],xy[2]),col=tmp.col[2], type="l", lty=3, lwd=1, ylab="", xlab="")

+ par(new=TRUE);

+ plot(y.upp0~x.tmp0,ylim=c(xy[3],xy[4]),xlim=c(xy[1],xy[2]),col=tmp.col[2], type="l", lty=3, lwd=1, ylab=yb[yi], xlab=sb[k])

+ rug(x.tmp0)

+ dev.off()

+

+ rm(tmp.ord,y.tmp0,x.tmp0,y.low0,y.upp0)

+ xy<-legLocate(x.tmp,wd[,1])

+ pngf1<-paste(ofname,yvar[yi],svar[k],"scatter.png",sep="_")

+ pdff1<-paste(ofname,yvar[yi],svar[k],"scatter.pdf",sep="_")

+

+ png(pngf1,width=720,height=560)

+ plot(y.tmp~x.tmp,ylim=c(xy[3],xy[4]),xlim=c(xy[1],xy[2]),col=tmp.col[1], type="p", pch=20, ylab="", xlab="")

+ par(new=TRUE);

+ plot(wd[,1]~x.tmp,ylim=c(xy[3],xy[4]),xlim=c(xy[1],xy[2]),type="p",pch=1,cex=0.5, ylab=yb[yi], xlab=sb[k])

+ dev.off()

+

+ pdf(pdff1,width=pdfwd, height=pdfht, family="Helvetica");

+ plot(y.tmp~x.tmp,ylim=c(xy[3],xy[4]),xlim=c(xy[1],xy[2]),col=tmp.col[1], type="p", pch=20, ylab="", xlab="")

+ par(new=TRUE);

+ plot(wd[,1]~x.tmp,ylim=c(xy[3],xy[4]),xlim=c(xy[1],xy[2]),type="p",pch=1,cex=0.5, ylab=yb[yi], xlab=sb[k])

+ dev.off()

+

+ } else {

+ if (is.na(parm[1])) {tmp.col<-rainbow(ncg);tmp.col1<-c("red","blue")} else {tmp.col<-rep("black",ncg);tmp.col1<-c("black","black")}

+ for (b in (1:ncg)) {

+ y00<-y.tmp[wd[,colvname]==colv.lv[b]]; x00<-x.tmp[wd[,colvname]==colv.lv[b]]

+ xy1<-legLocate(x00,y00)

+ pngf1<-paste(ofname,yvar[yi],svar[k],colvname,colv.lv[b],"smooth.png",sep="_")

+ pdff1<-paste(ofname,yvar[yi],svar[k],colvname,colv.lv[b],"smooth.pdf",sep="_")

+

+ png(pngf1,width=720,height=560)

+ plot(y00~x00,ylim=c(xy1[3],xy1[4]),xlim=c(xy1[1],xy1[2]),col=tmp.col1[1],type="p", pch=20, ylab=yb[yi], xlab=sb[k])

+ dev.off()

+

+ pdf(pdff1,width=pdfwd, height=pdfht, family="Helvetica");

+ plot(y00~x00,ylim=c(xy1[3],xy1[4]),xlim=c(xy1[1],xy1[2]),col=tmp.col1[1],type="p", pch=20, ylab=yb[yi], xlab=sb[k])

+ dev.off()

+

+ }

+ xy<-legLocate(x.tmp,y.tmp)

+ png(pngf,width=720,height=560)

+ for (b in (1:ncg)) {

+ y0<-y.tmp[wd[,colvname]==colv.lv[b]]; x0<-x.tmp[wd[,colvname]==colv.lv[b]]

+ if (b>1) par(new=TRUE)

+ plot(y0~x0,ylim=c(xy[3],xy[4]),xlim=c(xy[1],xy[2]),col=tmp.col[b], type="p", pch=px[b], ylab=yb[yi], xlab=sb[k])

+ }

+ legend(xy[5],xy[6],colv.lb,title=colvb, pch=px[1:ncg],bty="n",col=tmp.col)

+ dev.off()

+

+ pdf(pdff,width=pdfwd, height=pdfht, family="Helvetica");

+ for (b in (1:ncg)) {

+ y0<-y.tmp[wd[,colvname]==colv.lv[b]]; x0<-x.tmp[wd[,colvname]==colv.lv[b]]

+ if (b>1) par(new=TRUE)

+ plot(y0~x0,ylim=c(xy[3],xy[4]),xlim=c(xy[1],xy[2]),col=tmp.col[b], type="p", pch=px[b], ylab=yb[yi], xlab=sb[k])

+ }

+ legend(xy[5],xy[6],colv.lb,title=colvb, pch=px[1:ncg],bty="n",col=tmp.col)

+ dev.off()

+

+ png(pngf0,width=720,height=560)

+ for (b in (1:ncg)) {

+ y0<-y.tmp[wd[,colvname]==colv.lv[b]]; x0<-x.tmp[wd[,colvname]==colv.lv[b]]

+ tmp.ord<-order(x0); x00<-x0[tmp.ord]; y00<-y0[tmp.ord];

+ if (b>1) par(new=TRUE)

+ plot(y00~x00,ylim=c(xy[3],xy[4]),xlim=c(xy[1],xy[2]),col=tmp.col[b], type="l", lty=b, lwd=2, ylab=yb[yi], xlab=sb[k])

+ rm(tmp.ord,x00,y00)

+ }

+ legend(xy[5],xy[6],colv.lb,title=colvb,lty=(1:ncg),bty="n",col=tmp.col)

+ dev.off()

+

+ pdf(pdff0,width=pdfwd, height=pdfht, family="Helvetica");

+ for (b in (1:ncg)) {

+ y0<-y.tmp[wd[,colvname]==colv.lv[b]]; x0<-x.tmp[wd[,colvname]==colv.lv[b]]

+ tmp.ord<-order(x0); x00<-x0[tmp.ord]; y00<-y0[tmp.ord];

+ if (b>1) par(new=TRUE)

+ plot(y00~x00,ylim=c(xy[3],xy[4]),xlim=c(xy[1],xy[2]),col=tmp.col[b], type="l", lty=b, lwd=2, ylab=yb[yi], xlab=sb[k])

+ rm(tmp.ord,x00,y00)

+ }

+ legend(xy[5],xy[6],colv.lb,title=colvb,lty=(1:ncg),bty="n",col=tmp.col)

+ dev.off()

+

+

+ }

+ gg<-c(gg,"<td>",yb[yi]," vs. ",sb[k],"</br><a href=\"",pngf,"\" target=_BLANK><img src=\"",pngf,"\" width=320,height=320></a></td>")

+ }

+ return(gg)

+ }

> adjmean<-function(mdl, yi, xi) {

+ if (!is.na(xvname[1])) {allvname<- c(xvname,svname); all.lv<-c(xlv,slv);} else {allvname<-c(svname); all.lv<-slv;}

+ if (!is.na(colvname)) allvname<-c(allvname,colvname)

+ nv = length(allvname)

+ xi.lv <- levels(factor(WD[,svname[xi]]))

+ newd0 <- matrix(0,ncol=nv,nrow=length(xi.lv))

+ colnames(newd0)<-allvname

+ for (b in 1:length(all.lv)) {

+ if (all.lv[b]==0) {

+ newd0[,b]<-mean(WD[,allvname[b]],na.rm=TRUE)

+ } else {

+ uniqv <- unique(WD[,allvname[b]])

+ newd0[,b]<-uniqv[which.max(tabulate(match(WD[,allvname[b]], uniqv)))]

+ }

+ }

+ newd0[,svname[xi]]<-as.numeric(xi.lv)

+ if (!is.na(colvname)) {

+ for (b in 1:ncg) {

+ newd1<-newd0; newd1[,colvname]<-as.numeric(colv.lv[b]);

+ if (b==1) {newD<-newd1;} else {newD<-rbind(newD,newd1);}

+ }

+ f<-table(wd[,svname[xi]],wd[,colvname])

+ } else {

+ newD<-newd0; ncg<-1;

+ f<-table(wd[,svname[xi]])

+ }

+ pred<-predict(mdl, data.frame(newD),se.fit=TRUE)

+ meany.pop0<-tapply(wd[,yvname[yi]],wd[,svname[xi]],function(z) mean(z,na.rm=TRUE))[1]

+ if (ylink[yi]=="logit") meany.pop0<-log(meany.pop0/(1-meany.pop0));

+ if (ylink[yi]=="log") meany.pop0<-log(meany.pop0);

+ shift<-meany.pop0-pred$fit[1]

+ y.fit <- pred$fit+shift

+ y.low <- pred$fit+shift-pred$se.fit*1.96

+ y.upp <- pred$fit+shift+pred$se.fit*1.96

+ y.pred<- cbind(y.fit,y.low,y.upp);

+ tmp.ylab<-paste("Mean of", yb[yi]);

+ cname.pred<-c("Mean","Mean.low","Mean.upp")

+ if (ylink[yi]=="logit") {

+ y.pred<-exp(y.pred); y.pred<-y.pred/(1+y.pred);

+ tmp.ylab<-paste("% of", yb[yi]);

+ cname.pred<-c("Rate","Rate.low","Rate.upp")

+ }

+ if (ylink[yi]=="log") {

+ y.pred<-exp(y.pred); tmp.ylab<-paste("% of", yb[yi]);

+ cname.pred<-c("Rate","Rate.low","Rate.upp")

+ }

+ y.fit<-y.pred[,1]; y.low<-y.pred[,2]; y.upp<-y.pred[,3]

+ if (!is.na(xvname[1])) {

+ tmp.ylab<-paste("Adjusted", tmp.ylab)

+ cname.pred<-paste("adj.", cname.pred, sep="")

+ }

+ y.pred<-round(y.pred,dec)

+ colnames(y.pred)<-cname.pred

+ y.pred <-cbind(newD[,svname[xi]],y.pred); colnames(y.pred)[1]<-svname[xi]

+ if (!is.na(colvname)) {

+ y.pred<-cbind(newD[,colvname],y.pred); colnames(y.pred)[1]<-colvname

+ }

+ y.pred<-rbind(colnames(y.pred),y.pred)

+ px<-c(20,1:9)

+ if (is.na(parm[1])) {tmp.col<-rainbow(ncg);tmp.col1<-c("red","blue");

+ } else {tmp.col<-rep("black",ncg);tmp.col1<-tmp.col}

+

+ pngf<-paste(ofname,yvar[yi],svname[xi],"adjmean.png",sep="_")

+ pdff<-paste(ofname,yvar[yi],svname[xi],"adjmean.pdf",sep="_")

+

+ if (ncg>1) {

+ xy<-legLocate(newD[,svname[xi]],c(y.fit))

+ png(pngf,width=720,height=560)

+ for (b in 1:ncg) {

+ x.tmp<-newD[newD[,colvname]==colv.lv[b],svname[xi]]

+ y.tmp<-y.fit[newD[,colvname]==colv.lv[b]]

+ if (b>1) par(new=TRUE)

+ plot(y.tmp~x.tmp,ylim=c(xy[3],xy[4]),xlim=c(xy[1],xy[2]),col=tmp.col[b],type="b", pch=px[b],

+ ylab=tmp.ylab, xlab=sb[xi])

+ }

+ legend(xy[5],xy[6],colv.lb,title=colvb,pch=px[1:ncg],bty="n",col=tmp.col)

+ dev.off()

+

+ pdf(pdff,width=pdfwd, height=pdfht, family="Helvetica");

+ for (b in 1:ncg) {

+ x.tmp<-newD[newD[,colvname]==colv.lv[b],svname[xi]]

+ y.tmp<-y.fit[newD[,colvname]==colv.lv[b]]

+ if (b>1) par(new=TRUE)

+ plot(y.tmp~x.tmp,ylim=c(xy[3],xy[4]),xlim=c(xy[1],xy[2]),col=tmp.col[b],type="b", pch=px[b],

+ ylab=tmp.ylab, xlab=sb[xi])

+ }

+ legend(xy[5],xy[6],colv.lb,title=colvb,pch=px[1:ncg],bty="n",col=tmp.col)

+ dev.off()

+

+ for (b in 1:ncg) {

+ x.tmp<-newD[newD[,colvname]==colv.lv[b],svname[xi]]

+ y.tmp<-y.fit[newD[,colvname]==colv.lv[b]]

+ y.lci<-y.low[newD[,colvname]==colv.lv[b]]

+ y.uci<-y.upp[newD[,colvname]==colv.lv[b]]

+ xy<-legLocate(c(x.tmp,x.tmp),c(y.lci,y.uci))

+ png(paste(ofname,yvar[yi],svname[xi],colvname,colv.lv[b],"CI.png",sep="_"),width=720,height=560)

+ plotCI(x.tmp,y=y.tmp,li=y.lci,ui=y.uci,pch=20,lwd=1,col=tmp.col[1], xlim=c(xy[1],xy[2]),ylim=c(xy[3],xy[4]),

+ ylab=tmp.ylab, xlab=sb[xi], main=paste(colvb, colv.lv[b],sep=": "))

+ lines(x.tmp,y.tmp,lty=2)

+ dev.off()

+

+ pdf(paste(ofname,yvar[yi],svname[xi],colvname,colv.lv[b],"CI.pdf",sep="_"),width=pdfwd, height=pdfht, family="Helvetica");

+ plotCI(x.tmp,y=y.tmp,li=y.lci,ui=y.uci,pch=20,lwd=1,col=tmp.col[1], xlim=c(xy[1],xy[2]),ylim=c(xy[3],xy[4]),

+ ylab=tmp.ylab, xlab=sb[xi], main=paste(colvb, colv.lv[b],sep=": "))

+ lines(x.tmp,y.tmp,lty=2)

+ dev.off()

+

+ }

+ } else {

+ x.tmp<-newD[,svname[xi]]

+ xy<-legLocate(c(x.tmp,x.tmp),c(y.low,y.upp))

+ png(pngf,width=720,height=560)

+ plotCI(x.tmp,y=y.fit,li=y.low,ui=y.upp,pch=20,lwd=1,col=tmp.col[1], xlim=c(xy[1],xy[2]),ylim=c(xy[3],xy[4]),

+ ylab=tmp.ylab, xlab=sb[xi], main="Adjusted mean & 95% CI")

+ lines(x.tmp,y.fit,lty=2)

+ dev.off()

+

+ pdf(pdff,width=pdfwd, height=pdfht, family="Helvetica");

+ plotCI(x.tmp,y=y.fit,li=y.low,ui=y.upp,pch=20,lwd=1,col=tmp.col[1], xlim=c(xy[1],xy[2]),ylim=c(xy[3],xy[4]),

+ ylab=tmp.ylab, xlab=sb[xi], main="Adjusted mean & 95% CI")

+ lines(x.tmp,y.fit,lty=2)

+ dev.off()

+ }

+ oo<-c("</br>Adjusted mean ",yb[yi], " by ", sb[xi], "<table border=3>",mat2htmltable(y.pred),"</table>")

+ gg<-c("<td>",yb[yi]," vs. ",sb[xi],"</br><a href=\"",pngf,"\" target=_BLANK><img src=\"",pngf,"\" width=320,height=320></a></td>")

+ return(list(oo,gg))

+ }

>

> vlabelN<-(substr(vlabel,1,1)==" ");

> vlabelZ<-vlabel[vlabelN];vlabelV<-vlabel[!vlabelN]

> vnameV<-vname[!vlabelN];vnameZ<-vname[vlabelN]

> ny<-length(yvname); yb<-vlabelV[match(yvname,vnameV)]; yb[is.na(yb)]<-yvname[is.na(yb)]

> ns<-length(svname); sb<-vlabelV[match(svname,vnameV)]; sb[is.na(sb)]<-svname[is.na(sb)]

> ssf<-rep(",fx=FALSE", ns); ssf[sdf>0]<-paste(",k=",sdf[sdf>0],sep="")

> sxStr<-paste("s(",svname,ssf,sep="")

> sxStr[slv>0]<-paste("factor(",svname[slv>0],")",sep="")

> sxx<-paste("s(",svname,")",sep="")

> sxx[slv>0]<-paste("factor(",svname[slv>0],")",sep="")

> sxx<-matrix(sxx,ncol=1)

> if (!is.na(colvname)) {

+ sxStr[slv==0]<-paste(sxStr[slv==0],",by=factor(", colvname, ")",sep="")

+ sxStr[slv>0]<-paste(sxStr[slv>0],"*factor(", colvname, ")",sep="")

+ colv.lv<-levels(factor(colv)); ncg<-length(colv.lv); colvb<-vlabel[vname==colvname];

+ colv.lb<-vlabelZ[match(paste(colvname,colv.lv,sep="."),vnameZ)]

+ colv.lb[is.na(colv.lb)]<-colv.lv[is.na(colv.lb)]

+ colvb<-vlabelV[match(colvname,vnameV)]; if (is.na(colvb)) colvb<-colvname;

+ sxplots<-NA; sxterms<-NA

+ for (i in (1:ns)) {

+ sxplots<-c(sxplots,paste(svar[i],"_",colvname,colv.lv,sep=""));

+ sxterms<-rbind(sxterms,paste(sxx[i,],":factor(",colvname,")",colv.lv,sep=""))

+ }

+ sxplots<-sxplots[-1]; sxterms<-matrix(sxterms[-1,],ncol=ncg)

+ sxterms<-cbind(paste("factor(",colvname,")",sep=""),sxterms)

+ } else {ncg<-1;sxplots<-svar; sxterms<-sxx;}

> sxStr[slv==0]<-paste(sxStr[slv==0],")",sep="")

> nx<-0

> if (!is.na(xvname[1])) {

+ if (!is.na(colvname)) {xvname<-xvname[xvname!=colvname];}

+ nx<-length(xvname);

+ }

> if (nx>0) {

+ xb<-vlabelV[match(xvname,vnameV)]; xb[is.na(xb)]<-xvname[is.na(xb)];

+ xvv<-xvname; xvv[xlv>2]<-paste("factor(",xvname[xlv>2],")",sep="")

+ if (!is.na(colvname)) {

+ xvv[sxf=="S" | sxf=="s"]<-paste("factor(",colvname,")*",xvv[sxf=="S" | sxf=="s"],sep="")

+ }

+ xv1<-paste(xvv,collapse="+")

+ }

> if (is.na(par1)) par1<-1

> if (ny!=ns & par1==2) par1<-1

> if (par1==3) {nterms=ns*ncg*15+nx;} else {nterms=ncg*15+nx;}

> w<-c("<html><head>","<meta http-equiv=\"Content-Type\" content=\"text/html\" charset=\"gb2312\" /></head><body>")

> wtab<-"</br></br>Generalize additive models</br>"

> wpng<-"</br><table>";

> for (i in (1:ny)) {

+ if (par1!=3) {

+ wtmp<-"";

+ if (par1==2) {jstart<-i; jstop<-i;} else {jstart<-1; jstop<-ns;}

+ for (j in (jstart:jstop)) {

+ tmp.xx<-c(yvname[i],svname[j])

+ if (nx>0) tmp.xx<-c(tmp.xx,xvname)

+ if (!is.na(colvname[1])) tmp.xx<-c(tmp.xx,colvname)

+ wd<-WD[,tmp.xx];

+ wd<-wd[apply(is.na(wd),1,sum)==0,]

+ fml<-paste(yvname[i],"~",sxStr[j],sep="")

+ if (!is.na(colvname)) fml<-paste(fml,"+factor(",colvname,")",sep="")

+ if (nx>0) fml<-paste(fml,"+",xv1,sep="")

+ tmp.gam<-setgam(fml,i)

+ wtab<-c(wtab,paste("</br></br>Outcome:",yb[i]))

+ wtab<-c(wtab,paste("</br>Exposure:",sb[j]))

+ wtab<-c(wtab,gam2htmltable(tmp.gam))

+ if (slv[j]==0) {

+ wtmp<-c(wtmp,gam2pngs(tmp.gam,i,j))

+ } else {

+ stmp<-adjmean(tmp.gam,i,j)

+ wtmp<-c(wtmp,stmp[[2]])

+ wtab<-c(wtab,stmp[[1]])

+ }

+ }

+ wpng<-c(wpng,"<tr>",wtmp,"</tr>")

+ } else {

+ tmp.xx<-c(yvname[i],svname);

+ if (nx>0) tmp.xx<-c(tmp.xx,xvname)

+ if (!is.na(colvname[1])) tmp.xx<-c(tmp.xx,colvname)

+ wd<-WD[,tmp.xx];

+ wd<-wd[apply(is.na(wd),1,sum)==0,]

+ fml<-paste(yvname[i],"~",paste(sxStr,collapse="+"),sep="")

+ if (!is.na(colvname)) fml<-paste(fml,"+factor(",colvname,")",sep="")

+ if (nx>0) fml<-paste(fml,"+",xv1,sep="")

+ tmp.gam<-setgam(fml,i)

+ wtab<-c(wtab,paste("</br></br>Outcome:",yb[i]))

+ wtab<-c(wtab,gam2htmltable(tmp.gam))

+ if (sum(slv==0)>0) {

+ wpng<-c(wpng,"<tr>",gam2pngs(tmp.gam,i,0),"</tr>")

+ }

+ if (sum(slv>0)>0) {

+ for (k in 1:ns) {

+ if (slv[k]>0) {

+ stmp<-adjmean(tmp.gam,i,k)

+ wtab<-c(wtab,stmp[[1]])

+ wpng<-c(wpng,stmp[[2]])

+ }

+ }

+ }

+ }

+ }

> wpng<-c(wpng,"</table>")

> w<-c(w,wpng,wtab)

> w<-c(w,"</body></html>")

> fileConn<-file(paste(ofname,".htm",sep="")); writeLines(w, fileConn)

>

>

> proc.time()

”√ªß œµÕ≥ ¡˜ ≈

8.14 2.73 11.29
