# Supplementary material for: Accumulation of blood chromium and cobalt in the participants with metal objects: findings from the 2015 to 2018 National Health and Nutrition Examination Survey (NHANES)
Source: BMC Geriatr. 2023 Feb 3;23:72. doi: 10.1186/s12877-022-03710-3 (PMC9898935; doi:10.1186/s12877-022-03710-3)
Supplement: Supplementary file 4 — Additional file 4: Supplement Table 1. Some other characteristics of participants [file 12877_2022_3710_MOESM4_ESM.docx]

**Supplement Table 1** Some other characteristics of participants

|  | ***Without Metal Objects*** | ***With Metal Objects*** | ***p*** |
| --- | --- | --- | --- |
| Education level (%) |  | | 0.024 |
| Less than high school | 12.8% | 10.4% |  |
| High school | 24.2% | 27.0% |  |
| More than high school | 63.0% | 62.6% |  |
| Data Collection Years |  | | 0.149 |
| 2015-2016 | 22.4% | 20.5% |  |
| 2017-2018 | 77.6% | 79.5% |  |
| Marital |  | | 0.640 |
| Married / living with partner | 67.5% | 68.2% |  |
| Others | 32.5% | 31.8% |  |
| Tuna ^#^ |  | | <0.001 |
| No | 38.8% | 40.5% |  |
| Yes | 28.8% | 33.2% |  |
| Salmon ^#^ |  | | <0.001 |
| No | 40.5% | 42.2% |  |
| Yes | 27.0% | 31.4% |  |

^#^, variables with missing date as another category, the cumulation percent was not 100%;

% for categorical variables, P value was calculated by weighted chi-square test.
